# Prognostic Implications of Intratumoral and Peritumoral Infiltrating Lymphocytes in Pancreatic Ductal Adenocarcinoma

**DOI:** 10.3390/curroncol28060371

**Published:** 2021-11-01

**Authors:** Jung-Soo Pyo, Byoung Kwan Son, Hyo Young Lee, Il Hwan Oh, Kwang Hyun Chung

**Affiliations:** 1 Department of Pathology, Uijeongbu Eulji University Center, Eulji University School of Medicine, Uijeongbu-si 11759, Korea; jspyo@eulji.ac.kr; 2 Department of Internal Medicine, Uijeongbu Eulji University Center, Eulji University School of Medicine, Uijeongbu-si 11759, Korea; 2hyo0@eulji.ac.kr (H.Y.L.); 20180121@eulji.ac.kr (I.H.O.); kh.chung@eulji.ac.kr (K.H.C.)

**Keywords:** pancreatic ductal adenocarcinoma, tumor-infiltrating lymphocyte, immunoscore, prognosis, meta-analysis

## Abstract

This study aimed to elucidate the prognostic implications of intratumoral and peritumoral infiltrating T-lymphocytes in pancreatic ductal adenocarcinoma (PDAC) through a meta-analysis. A total of 18 eligible studies and 2453 PDAC patients were included in the present study. Intratumoral and peritumoral infiltrating lymphocytes were evaluated using various markers, such as CD3, CD4, CD8, FOXP3, and immune cell score. The correlations between these parameters and overall and disease-free survival were investigated and used in the meta-analysis. High intratumoral infiltration of CD3-, CD4-, and CD8-expressing lymphocytes was significantly correlated with better overall survival (hazard ratio (HR) 0.747, 95% confidence interval (CI) 0.620–0.900, HR 0.755, 95% CI 0.632–0.902, and HR 0.754, 95% CI 0.611–0.930, respectively). However, there was no significant correlation between PDAC prognosis and intratumoral FOXP3 or immune cell score (HR 1.358, 95% CI 1.115–1.655 and HR 0.776, 95% CI 0.566–1.065, respectively). Moreover, there was no significant correlation between the prognosis and peritumoral infiltrating T-lymphocytes. In evaluations of disease-free survival, only high intratumoral CD4 infiltration was correlated with a better prognosis (HR 0.525, 95% CI 0.341–0.810). Our results showed that high intratumoral infiltrating lymphocytes were significantly correlated with a better PDAC prognosis. However, among the tumor-infiltrating lymphocytes, CD3, CD4, and CD8 had prognostic implications, but not FOXP3 and immune cell score.

## 1. Introduction

Pancreatic ductal adenocarcinoma (PDAC) is one of the most aggressive malignant tumors [1,2]. Because PDAC shows aggressive growth and early metastasis, many patients are already in the advanced stage during diagnosis [1]. Therefore, targeted therapy for the individual PDAC patient may be needed. The development of biomarkers is useful in predicting therapeutic effect and prognosis [3]. PDAC frequently shows desmoplastic stroma with infiltrating inflammatory cells [4]. The tumor microenvironment, which is important in tumor behavior, comprises various cellular and molecular factors, including tumor-infiltrating lymphocytes (TILs) [5,6,7,8,9,10,11]. The compositions of TILs differ according to the tumor and tissue types [12,13]. CD8- and CD45-expressing T cells are significantly correlated with favorable survival in colorectal and ovarian cancers [12,13]. However, significant correlations between worse survival and regulatory T cells or myeloid-derived suppressor cells have also been reported [5,6,7,8,9,10,11]. In addition, these cells involve tumor growth and angiogenesis extension [5,6,7,8,9,10,11]. The interaction between TILs and stroma may be important in tumor progression [4]. In addition, because the diagnostic evaluation of pancreatic lesions can be limited, the diagnostic and prognostic implication of TILs can be useful. Nevertheless, detailed information on the prognostic implications of different TILs remain unclear in PDAC. In PDAC immunotherapy research, detailed information on TILs can be more important, and the prognostic implications according to TIL types can differ. Here, we investigated the prognostic impact of TILs in PDACs and performed a subgroup analysis based on TIL subtypes.

## 2. Materials and Methods

### 2.1. Published Study Search and Selection Criteria

Relevant articles were obtained by searching the PubMed and MEDLINE database on 30 September 2021. We used the following keywords: “pancreatic ductal adenocarcinoma” and “immunoscore or immune cell score or tumor-infiltrating lymphocyte or CD3 or CD4 or CD8 or FOXP3” and “prognosis or survival.” The titles and abstracts of all searched articles were screened for inclusion and exclusion. The included articles had information on the prognostic impact of TILs in PDAC. Included studies had a 60-month follow-up period at least. However, the number of patients was not included as an exclusion criterion. Non-original articles, such as case reports and review articles, were excluded. In addition, those not written in English were excluded from the present study.

### 2.2. Data Extraction

For the meta-analysis, data were obtained from 20 eligible studies [4,14,15,16,17,18,19,20,21,22,23,24,25,26,27,28,29,30,31,32]. The extracted data included the author’s information, study location, number of patients analyzed, markers for TILs in PDAC, and the correlation between TIL markers and PDAC survival. For the quantitative aggregation of survival results, the correlation between PDAC and survival was analyzed according to the hazard ratio (HR) using one of three methods. In studies that did not record the HRs or confidence intervals (CIs), we calculated these variables from the data using the HR point estimate, the log-rank statistic or its P-value, and the O-E statistic (the difference between the number of observed and expected events) or its variance. If these data were unavailable, the HR was estimated using the total number of events, number of patients at risk in each group, and the log-rank statistic or its P-value. Finally, if the only useful data were in the form of graphical representations of survival distributions, survival rates were extracted at specified times to reconstruct the HR estimate and its variance, under the assumption that patients were censored at a constant rate during the time intervals [33]. To reduce variability, the published survival curves were read independently by two authors. The HRs were then combined using Peto’s method [34]. The data associated with survival were extracted after a 60-month follow-up period. All data were obtained by two independent authors.

### 2.3. Statistical Analysis

The meta-analysis was performed using the Comprehensive Meta-Analysis software package (Biostat, Englewood, NJ, USA). The prognostic impacts of TILs were investigated and analyzed, dividing them into overall and disease-free survival. In addition, subgroup analyses based on intratumoral and peritumoral TILs were performed. Heterogeneity between the studies was checked using Q and I2 statistics, and was expressed as P-values. Additionally, a sensitivity analysis was conducted to assess the heterogeneity of eligible studies as well as the impact of each study on the combined effects. A random-effect model rather than a fixed-effect model was more suitable in the meta-analysis because the eligible studies used various populations. To assess publication bias, Begg’s funnel plot and Egger’s test were used; if it was significant, the fail-safe *N* and trim-fill tests were additionally used to confirm the degree of publication bias. The results were considered statistically significant at *p* < 0.05.

## 3. Results

### 3.1. Selection and Characteristics of the Studies

**Figure 1:**
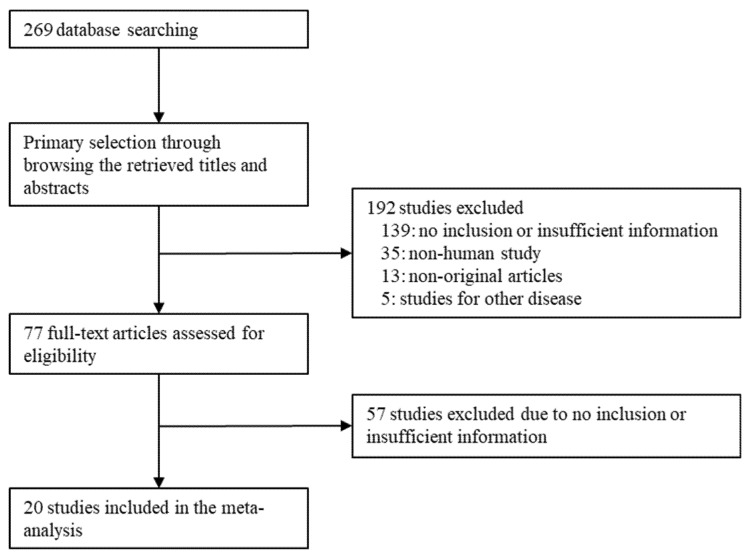
Flow chart showing the study search and selection methods.

We found 269 relevant articles during the primary search using the PubMed database. Upon screening and reviewing, we found that 196 articles had no information or had insufficient information for the meta-analysis. Among the remaining articles, 53 reports were excluded for the following reasons: non-human studies (*n* = 35), non-original articles (*n* = 13), and articles reporting other diseases (*n* = 5) (Figure 1). Finally, 20 eligible articles with a total of 2453 PDAC patients were included in the meta-analysis (Table 1).

### 3.2. Correlation between Intra- or Peritumoral Infiltrating T-lymphocytes and Overall Survival in Pancreatic Ductal Adenocarcinoma

The high intratumoral infiltration of CD3-, CD4-, and CD8-expressing T-lymphocytes was significantly correlated with better overall survival (HR 0.747, 95% CI 0.620–0.900, HR 0.755, 95% CI 0.632–0.902, and HR 0.754, 95% CI 0.611–0.930, respectively; Table 2). However, there was no significant correlation between the high peritumoral infiltration of CD3-, CD4- or CD8-expressing T-lymphocytes and overall survival (HR 1.029, 95% CI 0.847–1.251, HR 0.998, 95% CI 0.997–1.000, and HR 0.824, 95% CI 0.549–1.239, respectively; Table 3). By contrast, worse overall survival was significantly correlated with the high intratumoral infiltration of FOXP3-expressing T-lymphocytes (HR 1.358, 95% CI 1.115–1.655), but not with high peritumoral infiltration (HR 1.647, 95% CI 0.860–3.154). Moreover, there was no significant correlation between immune cell score and overall survival in PDAC (HR 0.776, 95% CI 0.566–1.065).

### 3.3. Correlation between Intra- or Peritumoral Infiltrating T-lymphocytes and Disease-Free Survival in Pancreatic Ductal Adenocarcinoma

The high intratumoral infiltration of CD4-expressing T-lymphocytes was significantly correlated with better disease-free survival (HR 0.525, 95% CI 0.341–0.810; Table 4). However, there was no significant correlation between the high intratumoral infiltration of CD3- or CD8-expressing T-lymphocytes and disease-free survival (HR 0.796, 95% CI 0.595–1.066 and HR 0.854, 95% CI 0.655–1.134, respectively). Moreover, there was no significant correlation between immune cell score and disease-free survival in PDAC (HR 0.766, 95% CI 0.558–1.052). However, there was no significant correlation between the high peritumoral infiltration of CD3- or CD8-expressing T-lymphocytes and disease-free survival.

## 4. Discussion

Generally, pancreatic cancers have worse survival rates [35]. Although the curative treatment is surgical resection, many pancreatic cancers cannot be treated due to late diagnosis [36]. Trials for immunotherapeutic treatment of PDACs have been performed to improve patient survival [37]. In addition, the patient’s prognosis is evaluated using limited information. TILs can be a valuable parameter in daily practice because the assessment is possible in various pathologic examinations, such as endoscopic ultrasonography-fine needle aspiration cytology. The clinical implications of TILs in pancreatic cancers have been reported [4,14,15,16,17,18,19,20,21,22,23,24,25,26,27,28,29,30,31,32]; however, various parameters have also been introduced for TILs in pancreatic cancers. Moreover, cumulative information for these varying TILs cannot be obtained from previous reports [4,14,15,16,17,18,19,20,21,22,23,24,25,26,27,28,29,30,31,32]. Hence, we studied the prognostic implications of various TIL markers and performed a detailed subgroup analysis of intra- and peritumoral T lymphocytes in PDACs through a meta-analysis.

Epithelial tumors, including pancreatic cancer, are composed of the tumor and surrounding stroma. Tumor invasion proceeds through the interaction with intra- and peritumoral stroma [38]. The peritumoral stroma comprises various components depending on stroma subtypes, including different types of lymphocytes [39]. T-lymphocytes can act in tumor suppression and promotion depending on their downstream pathways [40,41]. In preoperative evaluations, chronic inflammation of the pancreas can be confused as a malignancy [42]. Thus, the roles of TILs may be important in both intra- and peritumoral areas. TILs have been actively studied in gastrointestinal tumors, including colorectal cancers. However, in each cancer type, the implications of TILs may be different. Therefore, research is needed to improve the understanding of the implication of TILs in PDACs.

The present study analyzed the prognostic implications of TILs divided into CD3, CD4, CD8, FOXP3, and immune cell scores. In addition, prognosis was evaluated in terms of overall and disease-free survival. In the present meta-analysis, the most studied marker was CD8. Higher CD8-expressing T lymphocyte density in tumor tissue was significantly correlated with the expression of cytotoxicity genes of T lymphocytes of pancreatic cancer. [43,44]. In addition, abundant cytotoxic T lymphocytic infiltration was correlated with a better response to chemotherapy [45]. Evaluation of CD8-expressing TILs may be associated with not only the patient’s prognosis but also the therapeutic effect [46]. Hou et al. reported that patients with low CD8-expressing T cells and high levels of PD-L1 expression were correlated with worse survival [47]. In the present study, the high CD8-expressing T-lymphocytic group showed better overall survival than the low group. However, there was no significant difference in disease-free survival between the high and low groups. Subgroup analysis for the prognostic role of intratumoral infiltrating CD8-expressing T-lymphocytes was performed based on the TNM stage. In the subgroup including stage IV, the high intratumoral infiltration of CD8-expressing T-lymphocytes was significantly correlated with better overall survival (HR 0.777, 95% CI 0.654–0.923; data not shown). However, in the subgroup without stage IV, there was no significant correlation between CD8-expressing T-lymphocytic infiltration and overall survival (HR 0.695, 95% CI 0.478–1.012).

FOXP3-expressing regulatory T (Treg) cells might suppress anti-tumor immune response [48]. In addition, if Treg cells are removed, the anti-tumor immune response can be enhanced [48]. FOXP3-expressing regulatory T cells have also been correlated with clinical outcomes in other malignancies [4,49]. In previous studies, the prognostic implications of FOXP3 were controversial [50,51]. In the present study, PDACs with high FOXP3 TILs had worse overall survival than those with low FOXP3 TILs. However, this result was in contrast with the other TIL markers. In our meta-analysis, among the four studies with FOXP3, two studies showed a higher HR than 1. However, there was no statistical significance between the prognosis and FOXP3. Wang et al. reported a significant correlation between high FOXP3 expression and worse prognosis [30]. This discrepancy can result from the misinterpretation of heterogeneous FOXP3-expressed cells, such as functional Treg cells and non-Treg cells [48]. Although the prognostic implications differed between the included studies, in our meta-analysis, PDACs with high FOXP3 expression had worse prognoses than those with low FOXP3 expression (HR 1.358, 95% CI 1.115–1.655). This result was obtained from the interpretation of intratumoral FOXP3 expression. By contrast, there was no significant correlation between peritumoral FOXP3 expression and prognosis. Further studies are needed due to the results of one article with peritumoral FOXP3 expression.

The concept of the tumor microenvironment includes the interaction between tumor cells and the stroma in the peritumoral area. Tumor invasiveness is also closely related to the stroma of the peritumoral area. In the present study, we also evaluated the prognostic role of peritumoral lymphocytes. Peritumoral infiltrating lymphocytes were evaluated using CD3 and CD8 markers [4,14,15,16,17,18,19,20,21,22,23,24,25,26,27,28,29,30,31,32]. There was no significant correlation between peritumoral infiltrating lymphocytes and PDAC prognosis. From our results, the prognostic implications of intra- and peritumoral infiltrating lymphocytes were different. As described above, the prognostic implication of infiltrating lymphocytes was found only in the intratumoral area, but not in the peritumoral area. Moreover, the interpretation of the prognostic role of peritumoral infiltrating lymphocytes can be limited when patients previously have had pancreatitis. Further cumulative studies on peritumoral lymphocytes are therefore needed.

By definition, the immune cell score, which is known as an immunoscore in colorectal cancers, cannot be divided into intratumoral and peritumoral components. In the present study, the prognostic implications of immune cell score were investigated in PDACs. We have previously reported the correlation between high immunoscore and favorable prognosis in colorectal cancers [52]. The immune cell score is obtained by the summation of each score in the tumor center and the invasive margin. The markers of immunoscore in colorectal cancers are CD4- and CD8-positive TILs [53]. However, Tahkola et al. reported the prognostic implication of immune cell score using CD3 and CD8 markers instead [25,26]. In their studies, each score of the whole tumor area and the invasive margin for CD3 and CD8 were obtained from hot spots [25,26]. Nevertheless, there was no significant difference in overall and disease-free survival rates between PDACs with high and low immune cell scores. The immune cell score is evaluated in the tumor area, but not in the peritumoral area. In previous studies, TILs have been investigated using whole sections or tissue microarrays [25,26].

This study has some limitations. First, the evaluation of TILs is performed by investigating the hot spots or the whole tumor area. However, evaluating the whole tumor area cannot represent the overall tumor because all tumor areas are not produced as microscopic slides. The difference between studies with whole sections and hot spots could not be found in this study. Second, we evaluated TILs by dividing them into high and low groups. The impact of detailed stratification by the gradient of TILs, but not groups in PDAC, is unclear. However, a detailed analysis could not be performed due to insufficient information on eligible studies. Third, the prognosis of PDAC patients can be different by the chemotherapeutic effect. However, we could not measure the prognostic impact based on chemotherapy due to insufficient information. In addition, the change of TILs after chemotherapy could not be obtained. Fourth, subgroup analysis based on pancreatitis could not be performed due to insufficient information of eligible studies.

## 5. Conclusions

In conclusion, high TIL levels were significantly correlated with a better PDAC prognosis. Among the parameters of TILs, intratumoral CD3, CD4, and CD8, but not FOXP3 and immune cell score, have prognostic implications.

## Figures and Tables

**Table 1:** Main characteristics of the eligible studies.

References	Location	Number of Patients	Tumor Stage	No of Chemo-Radiotherapy	Analyzed Parameters
Diana 2016 [14]	Canada	145	I-III	126	CD8, FOXP3
Fukunaga 2004 [15]	Japan	80	I-IV	0	CD4, CD8
Homma 2014 [16]	Japan	22	I-III	17	CD8
Hwang 2016 [17]	Korea	30	I-III	0	CD4
Ino 2013 [4]	Japan	212	I-IV	94	CD4, CD8, FOXP3
Liu 2015 [18]	China	72	I-III	ND	CD8
Liu 2016 [19]	China	92	I-III	92	CD8
Michelakos 2020 [20]	USA	133	I-II	63	CD8
Mota Reyes 2019 [21]	Germany	74	I-IV	37	CD4
Nejati 2017 [22]	USA	136	I-IV	136	CD4
Sadozai 2021 [23]	Switzerland	112	I-III	ND	CD3
Seifert 2021 [24]	Germany	69	I-IV	17	CD3, CD4, CD8
Tahkola 2018 [25]	Finland	108	I-II	0	CD3, CD8, Immune cell score
Tahkola 2019 [26]	Finland	79	I-III	0	CD3, CD8, Immune cell score
Tang 2014 [27]	USA	228	I-IV	0	CD4, CD8
Tewari 2013 [28]	UK	81	I-III	ND	CD3, CD8
Tsukamoto 2019 [29]	Japan	235	I-IV	ND	CD8
Wang 2017 [30]	China	120	II	120	FOXP3
Wartenberg 2015 [31]	Switzerland	120	I-IV	120	CD8
Zhang 2019 [32]	China	305	I-III	0	CD3, CD8, FOXP3

ND, no description.

**Table 2:** Correlation between intratumoral infiltrating lymphocytes and overall survival in pancreatic ductal adenocarcinoma.

Tumor-Infiltrating Lymphocytes	Number of Subsets	Fixed Effect (95% CI)	Heterogeneity Test (*p*-Value)	Random Effect (95% CI)	Egger’s Test (*p*-Value)
Intratumoral CD3	5	0.747 (0.620, 0.900)	0.714	0.747 (0.620, 0.900)	0.138
Stage I to III	4	0.721 (0.590–0.880)	0.778	0.721 (0.590–0.880)	0.303
Western population	4	0.826 (0.648, 1.052)	0.917	0.826 (0.648, 1.052)	0.945
Eastern population	1	0.646 (0.482, 0.865)	1.000	0.646 (0.482, 0.865)	NA
Intratumoral CD4	6	0.755 (0.632, 0.902)	0.481	0.755 (0.632, 0.902)	0.424
Stage I to III	1	0.618 (0.261, 1.463)	1.000	0.618 (0.261, 1.463)	NA
Western population	3	0.774 (0.602, 0.994)	0.127	0.745 (0.515, 1.079)	0.361
Eastern population	3	0.736 (0.572, 0.949)	0.865	0.736 (0.572, 0.949)	0.736
Intratumoral CD8	12	0.804 (0.711, 0.910)	0.003	0.754 (0.611, 0.930)	0.053
Stage I to III	8	0.833 (0.699, 0.994)	<0.001	0.695 (0.478, 1.012)	0.035
Western population	5	0.786 (0.648, 0.953)	0.530	0.786 (0.648, 0.953)	0.452
Eastern population	7	0.817 (0.696, 0.959)	<0.001	0.709 (0.496, 1.013)	0.151
Intratumoral FOXP3	4	1.363 (1.133, 1.640)	0.338	1.358 (1.115, 1.655)	0.307
Stage I to III	3	1.397 (1.112, 1.755)	0.198	1.361 (1.010, 1.835)	0.238
Western population	1	0.965 (0.561. 1.660)	1.000	0.965 (0.561. 1.660)	NA
Eastern population	3	1.426 (1.172, 1.737)	0.448	1.426 (1.172, 1.737)	0.973
Immune cell score	2	0.776 (0.566, 1.065)	<0.001	0.776 (0.566, 1.065)	NA
Stage I to III	2	0.776 (0.566, 1.065)	<0.001	0.776 (0.566, 1.065)	NA
Western population	2	0.776 (0.566, 1.065)	<0.001	0.776 (0.566, 1.065)	NA

CI, Confidence interval; NA, Not applicable.

**Table 3:** Correlation between peritumoral infiltrating lymphocytes and overall survival in pancreatic ductal adenocarcinoma.

Tumor-Infiltrating Lymphocytes	Number of Subsets	Fixed Effect (95% CI)	Heterogeneity Test (*p*-Value)	Random Effect (95% CI)	Egger’s Test (*p*-Value)
Peritumoral CD3	4	1.001 (1.000, 1.002)	0.105	1.029 (0.847, 1.251)	0.912
Peritumoral CD4	1	0.998 (0.997, 1.000)	1.000	0.998 (0.997, 1.000)	NA
Peritumoral CD8	6	0.998 (0.997, 0.999)	< 0.001	0.824 (0.549, 1.239)	0.584
Peritumoral FOXP3	2	1.004 (1.001, 1.007)	0.135	1.151 (0.746, 1.775)	-NA

CI, Confidence interval; NA, Not applicable.

**Table 4:** Correlation between intra- and peritumoral infiltrating lymphocytes and disease-free survival in pancreatic ductal adenocarcinoma.

Tumor-Infiltrating Lymphocytes	Number of Subsets	Fixed Effect (95% CI)	Heterogeneity Test (*p*-Value)	Random Effect (95% CI)	Egger’s Test (*p*-Value)
CD3					
Intratumoral, high vs. low	3	0.796 (0.595, 1.066)	0.799	0.796 (0.595, 1.066)	0.699
Peritumoral, high vs. low	1	0.560 (0.290, 1.081)	1.000	0.560 (0.290, 1.081)	NA
CD4					
Intratumoral, high vs. low	2	0.525 (0.341, 0.810)	0.815	0.525 (0.341, 0.810)	NA
CD8					
Intratumoral, high vs. low	3	0.854 (0.644, 1.134)	0.955	0.854 (0.644, 1.134)	0.460
Peritumoral, high vs. low	2	0.810 (0.580, 1.131)	0.959	0.810 (0.580, 1.131)	NA
Immune cell score					
High vs. low	2	0.766 (0.558, 1.052)	0.915	0.766 (0.558, 1.052)	NA

CI, Confidence interval; NA, Not applicable.

## Data Availability

No new data were created or analyzed in this study. Data sharing is not applicable to this article.
